# Incidence of Infection of Enterovirus 71 and Coxsackieviruses A6 and A16 among Household Contacts of Index Cases in Dong Thap Province, Southern Vietnam

**DOI:** 10.1155/2020/9850351

**Published:** 2020-11-19

**Authors:** C. Q. Hoang, H. D. Nguyen, N. X. Ho, T. H. T. Vu, T. T. M. Pham, K. T. Nguyen, H. T. Nguyen, L. T. Hoang, H. Clapham, T. T. T. Nguyen, L. T. Phan

**Affiliations:** ^1^Directorial Board, Pasteur Institute in Ho Chi Minh City, Vietnam; ^2^Planning Division, Pasteur Institute in Ho Chi Minh City, Vietnam; ^3^Pasteur Institute in Ho Chi Minh City, Vietnam 167 Pasteur Street, District 3, Ho Chi Minh City, Vietnam; ^4^Training Center, Pasteur Institute in Ho Chi Minh City, Vietnam; ^5^Microbiology and Immunology Department, Pasteur Institute in Ho Chi Minh City, Vietnam; ^6^Medical Testing and Calibration Centers, Ho Chi Minh City Pasteur Institute, Ho Chi Minh, Vietnam; ^7^Saw Swee Hock School of Public Health, National University of Singapore, Singapore

## Abstract

**Background:**

Scarce information exists about immunity to hand, foot, and mouth disease (HFMD) among household contacts of index cases in Vietnam and what that means for reducing ongoing HFMD transmission in the community.

**Methods:**

We analyzed neutralizing antibodies (NT) and the incidence of enterovirus (EVs) infection among household contacts of index cases in a province where HFMD remains endemic. Throat swab and 2 mL blood samples from household contacts were collected at enrollment, during and after 2 weeks follow-up.

**Results:**

The incidence of EV-A71 infection among household contacts was 40/84 (47.6%, 95% Cl: 36.9-58.3%), compared with 106/336 (31.5%, 95% Cl: 26.6-36.5%) for CV-A6 and 36/107 (33.6%, 95% Cl: 24.7-42.6%) for CV-A16. The incidence of CV-A6 infection was fairly constant across ages; in contrast, CV-A71 and CV-A16 had some variation across ages. At baseline, higher geometric mean titer (GMT) of EV-A71, CV-A6, and CV-A16 antibody titers was found for 25-34-year groups (range 216.3 to 305.0) compared to the other age groups. There was a statistically significant difference in GMT values of CV-A6 and CV-A16 between those who had an infection or did not have infection among households with an index case of these serotypes.

**Conclusions:**

Our results indicated that adults were becoming infected with HFMD and could be contributing to the transmission. There is, therefore, a need for considering the household setting as an additional target for intervention programs for HFMD.

## 1. Introduction

Hand, foot, and mouth disease (HFMD) is a common pediatric illness that is of public health concern in the Asia Pacific region [1]. HFMD is characterized by brief febrile episodes and a characteristic skin rash with or without oral ulcers and is mainly a self-limited disease; however, in some cases, neurological complications can lead to serving outcomes such as brain-stem encephalitis, acute flaccid paralysis, aseptic meningitis, and even death [2, 3].

Enterovirus A71 (EV-A71), coxsackievirus A6 (CV-A6), CV-A10, and CV-A16, with CV-A6, are the main pathogens isolated from patients with clinically suspected HFMD in Vietnam [4–8]. EV-A71 is the main causative agent of severe and death cases occurring among HFMD cases in the southern provinces in Vietnam [4, 5, 8]. Until now, no specific treatment is available for HFMD [5, 8, 9]; therefore, there is a need for developing a cost-effective vaccine predominantly to prevent severe infections [3, 6]. Although a few companies from mainland China, Taiwan, and Singapore have successfully developed and licensed inactivated EV-A71 vaccines [2, 10–12], the scale of these vaccine use is still restricted, and the development of multivalent vaccines against HFMD is an ongoing effort.

HFMD is of the notifiable infectious diseases relied on symptomatic patients admitted to health care facilities [8, 13]. However, the proportion of asymptomatic cases remains quite high [14], perhaps acting as the sources of infection to naïve population. Taken together, suggesting that the current approach is unlikely to be adequate to control and prevention of HFMD in Vietnam. Therefore, the active surveillance system identifying the incidence of EVs infection rates and the immunity level among household contacts might contribute to decision-making on prevention strategies, especially HFMD vaccine programs.

In this study, we conduct a longitudinal study to better understanding of the incidence of EV infection rates and the immunity level among household contacts.

## 2. Materials and Methods

The study protocol was reviewed and approved by the institutional review board at the Pasteur Institute Ho Chi Minh City (PI HCMC), Vietnam (reference number: 02/PAS-HĐĐĐ). Written informed consent was obligatory for both patients and family members; parental informed consent was required on behalf of all children.

### 2.1. Sample Construction

The design and population of this longitudinal study have been published and described in detail previously [7]. Briefly, we recruited 150 patients aged under 15 who were clinically diagnosed with HFMD in Dong Thap Hospital and 581/600 household contacts who had close contact with the patient for 2 weeks before enrollment. All respondents were residents in Cao Lanh City and Cao Lanh District, Dong Thap Province.

### 2.2. Data and Specimen Collection

Before the study commencement, interviewers and healthcare staff completed a three-day training course on the study procedures, questionnaire, and specimen collection.

Respondents were informed of the study purposes, given informed consent forms. On the day of hospital admission, a face-to-face interview was performed to collect information regarding index cases' demographic, clinical, environmental, socioeconomic, behavioral, and epidemiological characteristics. Throat swabs for reverse transcription-polymerase chain reaction (RT-PCR) and 2 mL blood specimens for neutralizing antibody (NT) were then collected. After 1 day, eligible household contacts were recruited through home visits to collect relevant demographic, clinical, and behavioral information, and throat swabs and blood specimens are also collected, which were transported immediately at 4°C to the PI HCMC.

In the 2 weeks after the first index case and household contacts were enrolled, we followed up those cases and contacts through telephone interviews to ask about signs and symptoms. Once any cases reported HFMD signs or symptoms, those cases were invited to the hospital for physical examination and throat swabs for RT-PCR.

On day 14, 2 mL blood specimens for NT were collected at the hospital for both index cases and household contacts.

### 2.3. Sample Analysis

In this study, RT-PCR used previously published generic enterovirus (EVs) and EV-A71 primers [15]. In brief, viral RNA was extracted from throat swabs; then, a one-step multiplex RT-PCR assay was performed to detect EVs and EV-A71. After that, to classify EV genotypes or EV-A71 subgenogroups, all EVs and EV-A71 positive specimens were tested by using a combination of VP1 PCR and sequencing of the obtained PCR amplicon and previously published online tool [3, 16, 17].

In the present study, EV-A71 (subgenotypes B and C), CV-A6, and CV-A16 strains collected from HFMD cases in Southern Vietnam were used in a microneutralization assay to calculate neutralizing antibody titers for EV-A71, CV-A6, and CV-A16, in which the EV-A71 Genbank accession number (subgenotype B5_ KF557490; C4_KF557489; C5_KF557488), CV-A6 GenBank accession number (MT965681), and CV-A16 GenBank accession number (AM292441). The methods for measuring EV-A71 NT have been formerly described [4]. Briefly, plasma samples were diluted from 1 : 8 to 1 : 1024 and then were heat-inactivated at 56°C in 30 min. Diluted plasma and 100 TCID_50_ of the virus were pooled before incubating at 37°C for 1 hour and inoculating on human rhabdomyosarcoma cell lines (RD) (ATCC CCL136) in 96-well plate. Subsequently, these plates were incubated in a 5% carbon dioxide incubator at 37°C and observed for the progress of cytopathic effects (CPE) for 6-7 days. The neutralizing titer of a particular plasma was defined as the highest plasma dilution that resulted in the prevention of 50% CPE in the wells, and each dilution was tested in quadruplicate.

In the current study, seropositivity was defined as a NTtiter ≥ 1 : 8. An infection among household contacts was defined as those who had an RT-PCR positive swab or a 4-fold change or from negative to positive in a single EV-A71, CV-A6, and CV-A16 (or more) serotype neutralizing antibody titers between sample 1 and sample 2 [7, 18]. The incidence of HFMD infection was calculated by dividing the total number of new household contact infections for each serotype observed during a period of 2 weeks by the total members of households with an index case of that serotype for during the same time period [18].

### 2.4. Data Analysis

The sample's characteristics were summarized using frequency and proportion for categorical variables and mean and stand deviation or median and interquartile range for continuous variables.

The titers of NT were log-transformed to estimate the geometric mean titer (GMT) with 95% confidence intervals (Cl); all serums with NA titers of <8 were allocated a value of 4, and serums with NT titers of ≥1280 were allocated a value of 1280 [18]. Paired sample student's *t*-test was utilized to access the difference of GMTs.

Data were entered using Epi-Data version 3.1 (EpiData Association, Odense, Denmark), and all statistical analyses were performed in Stata version 13.0 (StataCorp, TX).

## 3. Results

Demographic, medical, environmental, socioeconomic, behavioral, and epidemiological characteristics have been published elsewhere [7]. In brief, for the 150 index cases who were clinically diagnosed with HFMD, the median age was 1.5 years (interquartile range (IQR) 0.2-5.2). 98% had fever, 85% had mouth sores, 53% had foot blisters, and 59% had hand blisters. 90/150 (60%) were RT-PCR positive, of which 78 cases were EVs (CV-A6 (57/78)) and 12 cases were EV-A71 (EV-A71 B5 (11/12)). Of 142 index cases for which we had two neutralizing antibody measurements, 101 (71%) had a ≥4-fold increase in neutralizing antibody titer of EV-A71, CV-A6, or CV-A16 across the two samples collected, wherein the NT against CV-A6, CV-A16, and EV-A71 were 61% (86/142), 18% (25/142), and 13% (19/142), respectively.

581/600 household contacts were recruited; the median age was 33 years (IQR 23-48). Among (6.2%, 36/581) symptomatic household contacts, 12 had RT-PCR-positive symptomatic infections, in which 10 cases were EVs (CV-A6, 6/10) and 2 cases were EV-A71 (EV-A71 B5, 2/2). 142/545 (26%) had a ≥4-fold increase in neutralizing antibody titer of EV-A71, CV-A6, or CV-A16 across the two samples collected, in which the NT against CV-A6, CV-A16, and EV-A71 were 19% (106/545), 7% (36/545), and 7% (40/545), respectively.

### 3.1. The Seroprevalence of Anti-EV-A71, CV-A6, and CV-A16 NT in Sample 1 and GMTs of Anti-EV-A71, CV-A6, and CV-A16 NT Neutralizing Antibodies

The percentage of household contacts who were EV-A71 seropositive at baseline sample was 77.8% (424/545), while 92.5% (504/545) were CV-A6 seropositive and 97.1% (529/545) were CV-A16 seropositive.

The CV-A16 NT seropositive rates were relatively constant across ages, which were no less than 74%, except for those aged in the 5 (48%) and 55-59 (62%) years old groups. Similarly, the seropositive rates of NT against EV-A71 among household contacts were also fairly constant across ages, but the figures were relatively low at 44%, 63%, 57%, and 57% in those aged less than 5, 10-14, 45-49, and >60 years old groups, respectively, while the CV-A6 NT seropositive rates tend to be declined in older groups, from 87% in the 5-9-year age groups to 65% in the 35-39-year age groups and 63% in the >60-year age group.

**Figure 1:**
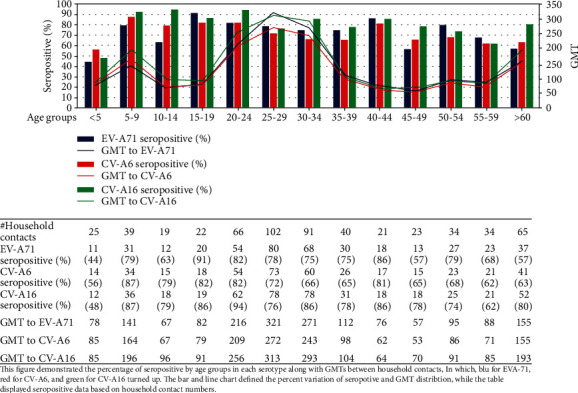
Seroprevalence of neutralizing antibodies against EV-A71, CV-A6, and CV-A16 by age group in sample 1 and geometric mean titers (GMTs) distribution in the positive serum samples.

The GMT of NT against CV-A16 was higher than those against CV-A6 and EV-A71 among household contacts aged less than 29 years. GMT values of all serotypes were the highest in the 20-34-year group than in other age groups (Figure 1). A significant declining trend in the GMT of NT against all serotypes was recorded from the peak in the 20-34 years old; the GMT for EV-A71, CV-A6, and CV-A16 dropped from 321, 272, and 313 among those aged 25-29 years to 122, 98, and 104 in those aged 35-39 years old, respectively.

### 3.2. The Infection Incidence of EV-A71, CV-A6, and CV-A16 among Household Contacts in the 2 Weeks Follow-Up

The incidence of EV-A71 infection among household contacts of an index cases infected with this serotype was 40/84 (47.6%, 95% Cl: 36.9-58.3%), compared with 106/336 (31.5%, 95% Cl: 26.6-36.5%) for CV-A6 and 36/107 (33.6%, 95% Cl: 24.7-42.6%) for CV-A16 (Table 1).

In terms of cross NT among various genotypes and various serotypes, the percentage of household contacts with NT against heterotypic virus varied between 2 and 3%. In detail, of 106 CV-A6 index cases, NT against CV-A16 and EV-A71 was reported in 3% (18/545) and 2% (9/545), respectively. Of 36 CV-A16 index cases, NT against CV-A6 and EV-A71 was reported in 3% (18/545) and 3% (17/545), respectively. Likewise, among 40 EV-A71 index cases, the percentage of NT against CV-A6 and CV-A16 was 2% (9/545) and 3% (17/545), respectively (Table 1).

### 3.3. Symptomatic Household Contacts

In the 2 weeks after household contacts were enrolled, 28/581 (5%), 11/581 (2%), 1/581 (0.2%), and 4/581 (0.7%) household contacts recorded illness and fever, HFMD, and a sore on hand, foot, or mouth, respectively.

In the present study, the majority of infections in household members were not accompanied by reported symptoms. 7.5% (3/40) of contacts who had an EV-A71 infection experienced illness in the 2 weeks follow-up; they were in the 25-29, 30-34, and 45-49 age groups. 1.9% (2/106) of CV-A6 infections experienced illness, and these were in the 25-29 years old and over 60 years old groups, and 2.8% (1/36) of CV-A16 infection experienced symptoms, with that one infection in the over 60 years old group.

### 3.4. Age-Dependent Infection Incidences of EV-A71, CV-A6, and CV-A16 among Household Contacts

The incidence of CV-A6 among contacts with an index case of CV-A6 was quite consistent across ages with no group less than 11%. This may be due to the larger numbers of index cases of CV-A6. In detail, the incidence of CV-A6 at around 33% (13/39), 27% (16/60), and 35% (19/54) for CV-A6 in household contacts aged 20-24, 25-29, and 30-34 years old compared with 38% (8/21), 35% (7/20), and 34% (11/32) in 50-54, 55-59, and more than 60 years old. For EV-A71 and CV-A16, the numbers in each age group are small, and we observed greater variation between the percentage of infected cases by age group. The incidences of EV-A71 and CV-A16 were highest at 100% among 20-24 and 5-9 years old groups, followed by CV-A6, 46% in those aged 40-44 years (Table 2).

The incidence of EV-A71 was consistently higher in those aged 20-39 years compared with CV-A6 and CV-A16. In the age group of 40-59 years, the incidences of EV-A71 and CV-A16 were similar and higher than those for CV-A6. There were no household contacts of 15-19-year group for EV-A71.

### 3.5. Relationship between GMTs, Infection, and Symptoms in Each Other for Serotype (EV-A71, CV-A6, CV-A16) among Infected Household Contacts

Among infected household contacts, the mean (± SD) GMT values of EV-A71 (*n* = 40) and CV-A16 (*n* = 36) were 2.77 (±0.69) and 2.54 (±0.23) in those had symptoms (*n* = 36) compared with 2.92 (±0.18) and 2.68 (±0.20) in those did not report symptoms, respectively. For CV-A6 (*n* = 106), the mean (± SD) GMT values of those who had symptom or not were 2.54 (±0.83) and 2.34 (±0.11), respectively. There was no significant difference in GMT values of EV-A71, CV-A6, and CV-A16 among those who had infections in those who had symptoms vs. those who did not (unpaired *t*-test; *P* > 0.05).

The mean baseline (± SD) GMT values of CV-A6 and CV-A16 in those who had no infection were 2.87 (±1.42) and 3.60 (±1.62), which was higher than in those who were infected, 2.27 (±1.03) and 2.34 (±0.75), respectively. There was statistically significant difference with regard to GMT values of CV-A6 and CV-A16 between those who had infection and no infection (unpaired *t*-test, *P* < 0.001). While the GMT values for EV-A71 were still higher in those who were not infected than those who had infection, but no significant difference in GMT values of EV-A71 was observed between these two groups (Table 3).

## 4. Discussion

The lowest levels of seropositivity of NT against EV-A71, CV-A6, and CV-A16 were found in household contacts aged less than the 5 years old group; only a half of those were seropositive, which are consistent with the previous studies [19–21]. We observed the highest NT levels in those aged 20-34 years old group, suggesting a more recent infection in this age group [22]. We also found that there was a gradually decline in the seropositivity among older adults. We show that household contacts were at high risk of infection; therefore, the need for screening strategy such as collecting specimens when household detected new cases may partially contributing to finding a substantial prevalence of HFMD that are currently undiagnosed in the current system.

We also found that the incidence of CV-A6 infection among household contacts was fairly constant across ages, while these figures for CV-A16 and EV-A71 varied across ages, perhaps due to smaller numbers of infections of these serotypes. Even though most cases are in children, infections causing a serological response are still occurring in older adults at similar rates to children, which in line with previous evidence showing that symptomatic HFMD was more common in vulnerable adults and young immunocompetent adults [23–26]. Of concern of clinical and public health viewpoint, a low proportion of adults with symptoms, but with a serological response, which could lead to the transmission of HFMD [25, 27, 28]. It is, therefore, necessary for household contacts and index cases to gain better understanding of HFMD transmission and how to halt the spread of HFMD among household contacts and community such as washing hands, staying at home, and seeking medical care when they were sick [7, 28, 29].

The household attack rates by serotype depend on pathogens, geographical locations, and sample size [22, 30, 31]. In our study, the household attack rate of EV-A71 was quite higher than CV-A6 and CV-A16, which was inconsistent with the results in Singapore [32], and in Baoji, northwest China [30]. It could be explained that stool samples, throat and rectal swabs, and swabs from vesicular fluid and oral ulcers from outpatients and inpatients were collected to estimate the estimated basic reproduction numbers in Singapore, while we only used throat swabs and blood specimens. Besides, the research population in Singapore was using surveillance data from outbreaks with only 33 individual outbreaks that were collected with more than 15 infected persons including childcare centers (18 months to 6 years) and kindergartens (4–6 years). Besides, the estimated basic reproduction numbers when the incubation period was varied from 3 to 7 days, while we recruited 150 index cases and 581 household contacts during 2 weeks follow-up, although Vietnam and Singapore are located at the tropical or subtropical zone with high temperature and high rate of rainfall and humidity. Two possible reasons could explain the difference in household attack rates in Baoji, northwest China. Firstly, the climates are different seasonal temperature and rainfall. Second, the total figures for daily new cases in Baoji city were used, and the duration of the exponential growth phase of the outbreak is more than two months in the scale of the whole city in Baoji [30]. Because of these reasons mentioned above, the household attack rates by serotypes estimated in our study presented quite great differences as studies observed in Baoji, northwest China, and Singapore.

In the present study, 6.20% (36/581) household contacts exhibited symptoms. Among infections, less than 10% of EV-A71 infection had symptoms in the 25-29, 30-34, and 45-49 age groups and among the 25-29-, 50-54-, and over 60-year groups for CV-A6 and CV-A16. These proportions of symptoms were in agreement with a previous study, approximately 11% of exposed adults get infected and less than 1% manifest the disease [27], possibly relating to EVs exposure leading to immunologic memory [33].

In this present study, we found the overall seroprevalence rates of anti EV-A71, CV-A6, and CV-A16 antibody were quite high, 77.8%, 92.5%, and 97.1%, respectively. The lower seroprevalence rate of EV-A71 across all ages could suggest that household contacts in this study are the most susceptible to EV-A71 infection and suggest the risk of an outbreak of EV-A71 in the coming years [34].

The higher GMT of NT against CV-A16 compared with those against CV-A6 and EV-A71 in the household contacts age group of less than 29 years could be explained by its recent emergence. The lower GMT for CV-A6 and EV-A71 suggests these serotype infections were uncommon during the study period, and reinfection of the household contacts is sporadic [34, 35].

In this study, we found that the GMT for EV-A71 was higher in those who were not infected, but there was no significant difference in GMT values, though the numbers were small. However, the higher GMT levels of CV-A6 and CV-A16 in those who had no infection seen during the study period were more likely to mild infection resulting in underreporting [36]. Our findings suggest that for CV-A6 and CV-A16, higher levels of NT lead to sterilizing immunity, whereas at lower levels, infection could still occur. However, we did not observe any difference in NT among those infected whether they showed symptoms or not.

Recent evidence reported on recurrent HFMD episodes resulting from heterotypic reinfection in and lack of cross-neutralization among these four enterovirus serotypes observed in vaccine studies [37–40]. In the previous study showed that seropositive rates for heterotypic viruses (EV-A71, CV-A6, CV-A16), while at follow-up, were only a small proportion (3%–23%) of the patients had seroconverted for heterotypic viruses [41], which was higher than our study. However, it indicated that EV-A71, CV-A6, and CV-A16 cross-neutralization is absent or occurs in only a limited proportion of patients [42, 43]. But, it cannot be ruled out that these rates of seropositive household contacts and seroconversion are due to prior exposure or coinfection with other serotypes that PCR may not have identified [41].

The low proportion of infections with symptoms has been described among adults in previous studies [25, 27, 28]; our results indicated that adults were becoming infected with HFMD due to EV-A71, CV-A6, and CV-A16 infections that were highly prevalent among household contacts [35], which could mean that they are contributing to transmission. However, we suggested further work is needed to establish whether these antibody rises were combined with infectiousness. Thus, screening syndromic, collecting specimens, and testing approach for preventing HFMD transmission are highly recommended and included in routine and sentinel surveillance systems for physicians and healthcare staff to better manage HFMD for household contacts of index cases in Vietnam. Our findings suggest there is a need for considering the household setting as an additional target for intervention programs for HFMD.

## 5. Conclusion

Our results indicated that adults were becoming infected with HFMD and could be contributing to the transmission. There is, therefore, a need for considering the household setting as an additional target for intervention programs for HFMD.

## Figures and Tables

**Table 1:** Laboratory characteristics and incidences of EV-A71, CV-A6, and CV-A16 infection among household contacts of index cases (*n* = 545).

Variables	EV-A71	CV-A6	CV-A16
Seropositive	424	504	529
Infection	40	106	36
RT-PCR	02	06	0
RT-PCR and seroconversion	01	05	0
Seroconversion	40	106	36
EV-A71 NT against	—	09	17
CV-A6 NT against	09	—	18
CV-A16 NT against	17	18	—
Number of household contacts among households with an index case of each serotype	84	336	109
Incidence rate	47.6% (40/84)	31.5% (106/336)	33.0% (36/109)

**Table 2:** Age-dependent incidences of EV-A71, CV-A6, and CV-A16 infection among household contacts.

Age group	EV-A71			CV-A6			CV-A16	
	Infected	Members	%	Infected	Members	%	Infected	Members	%
<5	1	4	25	2	12	17	0	5	0
5-9	1	3	33	9	21	43	2	2	100
10-14	1	4	25	5	12	42	2	4	50
15-19	2	*£*	—	3	14	21	0	4	0
20-24	9	9	100	13	39	33	8	12	67
25-29	6	12	50	16	60	27	8	27	30
30-34	6	14	43	19	54	35	3	12	25
35-39	2	7	29	5	19	26	1	7	14
40-44	0	2	0	6	13	46	0	4	0
45-49	2	6	33	2	19	11	1	3	33
50-54	6	9	67	8	21	38	5	8	63
55-59	2	4	50	7	20	35	3	6	50
>60	2	10	20	11	32	34	3	13	23

£: index case was not infected with EV-A71.

**Table 3:** Relationship between GMTs, symptoms, and infection (EV-A71, *n* = 24; CV-A6, *n* = 82; CV-A16, *n* = 16) in each serotype among households with an index case of these serotypes.

Variables	GMT_EV-A71	GMT_CV-A6	GMT_CV-A16
Symptoms*^┴^*			
Yes	0.22 ± 0.15	0.25 ± 0.43	0.33 ± 0.21
No	0.29 ± 0.05	0.15 ± 037	0.14 ± 0.35
*P* value	0.660	0.314	0.197
Infection^≠^			
Yes	2.92 ± 1.12	2.27 ± 1.03	2.34 ± 0.75
No	3.25 ± 1.67	2.87 ± 1.42	3.60 ± 1.62
*P* value	0.299	<0.001	<0.001

^┴^Experienced illness in the past two weeks. Fever: experienced fever in the past two weeks; HFMD: experienced hand, foot, and mouth disease in the past four weeks; rash: exhibited a sore on hand, foot, or mouth in the past 4 weeks. ^≠^Unequal variances.

## Data Availability

The data used to support the findings of this study are available from the corresponding author upon request.

## Abbreviations

CV: Coxsackieviruses
EVs: Enteroviruses
EV-A71: Enterovirus A71
CV-A6: Coxsackievirus A6
CV-A16: Coxsackievirus A16
HFMD: Hand foot and mouth disease
GMT: Geometric mean titer
NT: Neutralizing antibody
RT-PCR: Reverse transcription-polymerase chain reaction.
